# Reducing care-resistant behaviors during oral hygiene in persons with dementia

**DOI:** 10.1186/1472-6831-11-30

**Published:** 2011-11-19

**Authors:** Rita A Jablonski, Ann Kolanowski, Barbara Therrien, Ellen K Mahoney, Cathy Kassab, Douglas L Leslie

**Affiliations:** 1 CRNP, The Pennsylvania University School of Nursing, 201 Health & Human Development East, University Park, PA 16802, USA; 2 School of Nursing, The Pennsylvania State University, University Park, Pa. 16802, USA; 3 University of Michigan School of Nursing 400 North Ingalls Building Room 2162 Ann Arbor, MI 48109-0482, USA; 4 William F. Connell School of Nursing, Boston College, 140 Commonwealth Avenue, Chestnut Hill, MA 02467, USA; 5 By The Numbers 530 Hartman Road, West Decatur, PA 16878, USA; 6 The Pennsylvania State University, College of Medicine, A210 Public Health Sciences Hershey Medical Center, USA

## Abstract

**Background:**

Nursing home residents with dementia are often dependent on others for mouth care, yet will react with care-resistant behavior when receiving assistance. The oral health of these elders deteriorates in the absence of daily oral hygiene, predisposing them to harmful systemic problems such as pneumonia, hyperglycemia, cardiac disease, and cerebral vascular accidents. The **purpose **of this study is to determine whether care-resistant behaviors can be reduced, and oral health improved, through the application of an intervention based on the neurobiological principles of threat perception and fear response. The intervention, called Managing Oral Hygiene Using Threat Reduction, combines best mouth care practices with a constellation of behavioral techniques that reduce threat perception and thereby prevent or de-escalate care-resistant behaviors.

**Methods/Design:**

Using a randomized repeated measures design, 80 elders with dementia from 5 different nursing homes will be randomized at the individual level to the experimental group, which will receive the intervention, or to the control group, which will receive standard mouth care from research team members who receive training in the proper methods for providing mouth care but no training in resistance recognition or prevention/mediation. Oral health assessments and care-resistant behavior measurements will be obtained during a 7-day observation period and a 21-day intervention period. Individual growth models using multilevel analysis will be used to estimate the efficacy of the intervention for reducing care-resistant behaviors in persons with dementia, and to estimate the overall efficacy of the intervention using oral health outcomes. Activity-based costing methods will be used to determine the cost of the proposed intervention.

**Discussion:**

At the conclusion of this study, the research team anticipates having a proven intervention that prevents and reduces care-resistant within the context of mouth care. Long-term objectives include testing the effect of the intervention on systemic illnesses among persons with dementia; examining the transferability of this intervention to other activities of daily living; and disseminating threat reduction interventions to nursing home staff, which may radically change the manner in which care is provided to persons with dementia.

**Trial Registration:**

ClinicalTrials.gov: NCT01363258

## Background

Older adults residing in nursing homes (NHs) experience difficulty maintaining good oral health[1-4]. Oral health in NHs has been described as "deplorable,"[[5](p251)] with evidence that "a high proportion of elderly nursing home residents suffer from poor oral hygiene and oral health neglect."[[4](p100)] The need for good oral health is significant with older adults for a variety of reasons. The majority of NH residents arrive dentate[6]. Older adults experience faster plaque production than younger adults because of the dual effects of gingival recession and reduced saliva production[7]. Poor oral hygiene causes periodontal disease which in turn creates tooth loss. The remaining teeth shift, causing loss of occlusal surfaces and subsequent chewing and swallowing problems. These problems place older adults at risk for malnutrition[8]. Other systemic diseases associated with poor oral hygiene include aspiration pneumonia, [9] diabetes, [10,11] and coronary artery disease [12-14].

The need for good oral hygiene is complicated by the dependence many NH residents have on others to provide basic care. Most require assistance in at least one activity of daily living while more than half are dependent on others for all activities of daily living, including mouth care[15]. In addition to dependence, some persons with dementia exhibit care-resistant behavior (CRB) during helping interactions. CRBs are actions "invoked by a caregiving encounter...defined as the repertoire of behaviors with which persons with dementia withstand or oppose the efforts of a caregiver."[[16](p28)]. In previous research CRBs were categorized as "uncooperative behavior,"[17-19]" "disruptive behavior," [20-22] and "agitation." [23] The progression of dementia coupled with an increased need for assistance increases the likelihood of CRB on the part of the NH resident [23,24].

Eighty percent of certified nursing assistants (CNAs) have reported experiencing CRBs while providing mouth care [25]. These behaviors ranged from mild resistance (e.g. clenching mouths closed or turning the head away) to extreme resistance (e.g., hitting or kicking the CNA) [26]. CRBs were often triggered by the CNA performing mouth care instead of allowing the older adult to do so [27]. Other precipitants to CRBs during mouth care included caregivers attempting to forcefully insert the toothbrush or swab into residents' mouths without alerting them; lack of praise or encouragement; compound commands versus simple one-step commands; no smiling or positive facial cues from the caregiver; and attempting to provide mouth care without prompts or gestures[27]. These findings support the theoretical foundation of this study, that is, CRB is a fear-evoked response to caregivers' unintentionally threatening behavior during mouth care.

As discussed in detail in another publication [28], CRB is conceptualized as behavioral responses to a perceived threat. In other words, the older adult with dementia sees the caregiving actions as a form of assault. This conceptualization of CRB is based on the neurobiology of the limbic system. The limbic system is designed to detect threat and initiate protective fear responses of freeze, flight, or fight [29-31]. The primary structure is the amygdala, comprised of varied sets of nuclei: lateral, basal, basolateral, and basomedial[29]. These nuclei interface with other structures in the limbic system, primarily the hippocampus, as well as the brainstem[32]. In a healthy brain, the hippocampus and select areas of the cerebral cortex receive and process signals from the amygdala to provide awareness, context, and judgment to the threat perception and subsequent fear response[30,33]. When the brain, especially the cerebral cortices and hippocampus, is compromised by the pathology of dementia, the ability to apply context to the perceived threat deteriorates. As reasoning and perception become altered, persons with dementia may interpret non-threatening situations (such as a CNA attempting to help the elder with mouth care) as an actual assault[33,34].

This protocol is innovative in specifically addressing threat perception and fear responses in individuals with dementia who are unlikely to have sufficient contextual or cognitive control over threat perceptions due to altered neurological structures and functions. The specific strategies proposed here are expected to reduce perceptions of mouth care and caregiver as threatening and, in turn, limit or prevent CRBs associated with automatic and reflexive fear responses.

In summary, there is a tremendous gap in knowledge regarding interventions designed to prevent and reduce CRB during mouth care in order to improve the oral health of persons with dementia. Persons with dementia and CRB have been systematically excluded from intervention studies developed to improve oral health among NH residents, even though older adults with moderate to severe dementia have worse oral health those with no or mild dementia. Interventions to address oral health outcomes for NH residents with moderate to severe dementia, and who resist care, have not been systematically examined in either nursing or dental research studies. In order to improve nursing clinical practice and to address oral health disparities, it is imperative to design studies that focus on these interventions.

The purpose of this study is to determine whether CRBs can be reduced, and oral health improved, through the application of an intervention based on the neurobiological principles of threat perception and fear response. **The primary specific aims of the study are to:**

1. Evaluate the efficacy of the Managing Oral Hygiene Using Threat Reduction (MOUTh) intervention for reducing CRBs in persons with dementia;

2. Validate the overall efficacy of the MOUTh intervention using nurse-sensitive oral health outcomes-- swollen and bleeding gums, cleanliness of the oral cavity, saliva, and integrity of the lips and oral mucosa; and

3. Calculate the cost of the MOUTh intervention.

The hypotheses based on the specific aims are as follows:

H1. Implementation of the MOUTh intervention will significantly reduce CRBs during mouth care compared to usual mouth care;

H2. The MOUTh intervention will improve oral health in older adults with dementia through the resolution of swollen and bleeding gums, improved cleanliness of the oral cavity, and the resolution of dry, cracked, and fissured oral mucosa and lips compared to usual care.

## Methods/Design

A randomized repeated measures design will be used in this clinical trial. NH residents will be randomized at the individual level to the experimental group, which will receive the intervention, or to the control group, which will receive standard mouth care from research team members who receive training in the proper methods for providing mouth care but no training in resistance recognition or resistance prevention/mediation. For baseline data collection, the behavior raters (research assistants trained to observe and record CRB) will observe both control and experimental NH residents receiving mouth care from the NH staff twice daily (AM: after breakfast but before lunch and PM: immediately after the evening meal) for 7 days. Members of the experimental and control groups will then receive mouth care from interventionists (research team members trained to provide mouth care) twice daily for 21 days, using the same schedule. A 21-day intervention period provides the greatest opportunity for obtaining the maximum benefit from the MOUTh intervention. Oral health outcome data will be collected 5 times: once prior to the 7-day observation period; once after the 7-day observation period but before the initiation of the intervention; after 7 days of intervention; after 14 days of intervention; and after 21 days of intervention. This schedule, which is depicted in Table 1 is designed to fully capture changes in oral health throughout the progression of the study.

**Table 1:** Intervention & Data Collection Schedule

	Steps & Measures	Frequency
Recruitment	**Screen and Consent** Written consent from responsible party Examination of medical records to confirm inclusion/exclusion criteria Examination of NH resident for ability to hold toothbrush, raise hand to mouth MMSE Resnick et al.'s Evaluation to Sign Consent if MMSE 18 or higher Written consent from NH resident if 6/8 questions answered correctly on Resnick et al.'s Evaluation to Sign Consent	Once

Baseline, pre-observation	**Randomization (Experimental or Control) and Baseline** Baseline Chart Review (demographic data, co-morbidities) Global Deterioration Scale (GDS) Katz Activities of Daily Living (ADL) Oral Health Assessment Tool (OHAT)	Once Once Once

Days 1-7 Baseline Observations Phase	**Experimental and Control Group** Resistiveness to Care (RTC-r) OHAT Surveillance for acute infections Surveillance for psychoactive medications	Twice daily × 7 days Once, after Day 7's mouth care session Once on Day 7 for previous 7 days Once on Day 7 for previous 7 days

Intervention Phase Days 8-28	**Experimental Group** Mouth Care per MOUTh protocol RTC-r OHAT Surveillance for acute infections Surveillance for psychoactive medications **Control Group** Mouth Care per MOUTh protocol RTC-r OHAT Surveillance for acute infections Surveillance for psychoactive medications	Twice daily × 21 days Twice daily × 21 days Three times, once after Day 14's evening mouth care session, once after Day 21's evening mouth care session, and once after Day 28's evening mouth care session Three times, once on Days 14, 21, and 28 for preceding week Three times, once on Days 14, 21, and 28 for preceding week Twice daily × 21 days Twice daily × 21 days Three times, once after Day 14's evening mouth care session, once after Day 21's evening mouth care session, and once after Day 28's evening mouth care session Three times, once on Days 14, 21, and 28 for preceding week Three times, once on Days 14, 21, and 28 for preceding week

### Description of the MOUTh Intervention

The MOUTh intervention was developed and pilot-tested in a previous study[35]. It contains three components: best mouth care practices for older adults with natural dentition and dentures[36-40]; recognition of CRB[16,41]; and strategies to reduce threat perception during the provision of mouth care[35,36,40,42-44]. The mouth care protocol integrates best mouth care practices with threat reduction strategies derived from the neurobiological, nursing, and dental literature. For example, best mouth care practices included using warm water for rinsing and using interdentate brushes for flossing[36-40]. Threat reduction strategies are a constellation of techniques congruent with previously cited neurobiological and nursing studies. These techniques, which were culled from extant literature as well as from techniques that were successfully employed in a previously published pilot study[35], include interventionist behaviors designed to minimize threat appraisal, such as smiling and relaxed demeanor,[45] distraction, bridging (having an elder hold a toothbrush while the interventionist brushes the elder's teeth), and cueing (the use of polite, one-step commands)[40,42]. Interventionists are trained to encourage the elder to do as much of his or her mouth care as possible. The rationale for this technique is that self-care is unlikely to be perceived as threatening[45]. Experimental interventionists are also trained to avoid the use of "elderspeak," a term used to describe "baby talk" speech patterns associated with infants and pets but inappropriately employed when engaged with older adults: high pitch, short sentences, sing-song cadence, patronizing tone, use of collective pronouns, and infantilizing terms (baby, honey, dearie)[43,44]. Elderspeak is a documented trigger to CRB[43,44] because its dehumanizing approach heightens threat perception.

### Description of Usual Care

Usual mouth care is a challenging issue. For many persons who resist care, "usual" mouth care may mean NO mouth care[27]. Designing a study in which a potentially powerful intervention is compared to an absence of care would be meaningless. In order to have parity between the control and experimental groups, members of both will receive mouth care from interventionists from the research team. The difference will be that the control interventionists will NOT receive training in techniques designed to prevent and reduce CRB. Instead, the control interventionists will receive training separate from the experimental interventionists. That training will consist of best mouth care practices specific to older adults[36-40]. For consistency, both subjects in the control and experimental groups will receive the same mouth care supplies during the duration of the study: Biotene™ toothpaste (dentate); Biotene™ alcohol-free mouthwash (both); soft toothbrushes (dentate); denture brushes (dentures); interdentate sticks (dentate); denture cleansing paste (denture); and denture cups.

### Setting

Five Pennsylvania NHs that vary in size, ownership, reimbursement patterns, and location will be used in order to provide a large population for recruitment, afford diversity, and enhance the generalizability of the findings for future translational research. The NHs range in size from 159 beds to 404 beds, with a total number of 1,355 beds. All but one have a segregated dementia unit, ranging in size from 30 beds to 60 beds, for a total of 143 beds. Based on previous pilot data[35] and information provided by the NH administration, approximately 20% of the sample of 1,355 potential subjects meets the inclusion criteria, resulting in a recruitment pool of 270 NH residents from which the final sample of 60 NH subjects will be obtained.

### Recruitment, Sample, Sample Size

Power analyses were conducted in order to determine the sample size needed to provide sufficient power (1-β > .80) for assessing relationships between the explanatory variables and resistive behaviors and oral health outcomes. Data from the pilot study informed these analyses[35]. The power analyses were conducted using the software PINT[46], which is based on the work of Snijders and Bosker[47,48]. PINT estimates standard errors for regression coefficients in a two-level model, from which power can be calculated for a given effect size and significance level (α). Effect size was taken to be the absolute value of a standardized regression coefficient, and α was set at 0.05. Because PINT is designed for a two-level model rather than a three-level model, the second and third levels (participant and nursing home) were combined for power analysis purposes. Inputs into PINT include the total number participants to be sampled across all nursing homes (*N*) and the number of data collection times (*n*) per participant. Given *N *and *n*, the total sample size is *N *× *n*. Plausible effect sizes and other required inputs into PINT were based on the pilot study[35].

Based on results from the pilot study,[35] it is plausible to expect that the intervention group-control group dummy variable will have an effect size of 0.05 or larger in both the models for care resistive behaviors and the models for oral health outcomes. Power will then be high (1-β > 0.90) provided there are 42 observations per participant (twice daily for 21 days, so that *n *= 42), 12 participants across both the intervention and control groups within each nursing home (assuming 16 participants recruited, less 25% attrition), and five nursing homes (so that *N *= 12 × 5 = 60). This corresponds to a sample size of 2,520 (*N *× *n *= 42 × 60 = 2,520). If the effect size is somewhat larger (0.1), power is extremely high (1-β ≈ 1). In summary, given a 25% expected attrition rate, 80 participants should be recruited for the study (40 in intervention and 40 in control groups).

Subjects will be recruited according to the following **inclusion criteria: **English-speaking; age 65 or older; documented diagnosis of dementia, Alzheimer's disease, vascular dementia, or Lewy body dementia; identified by NH staff as resistant to mouth care; at least 2 adjacent teeth AND/OR daily wearing of at least one denture plate; the ability to hold a toothbrush; and the ability to move his or her hand to his or her mouth. Once subjects are identified, consent will be obtained initially from the responsible party. After consent is obtained, subjects will be tested using the Mini-mental Status Examination (MMSE)[49]. Those with scores 18 or higher will be evaluated for the capacity to sign a consent form using a modification of Resnick et al.'s Evaluation to Sign Consent instrument[50]. This evaluation includes 8 questions that assess understanding of the study. Participants with dementia must score at least 6 out of the 8 questions correctly in order to sign the consent form. If the elder is deemed unable to sign the consent form assent will be obtained during each interaction between the NH resident and any member of the research team. **Exclusion criteria **are: age less than 65; no documented diagnosis of dementia; inability to hold a toothbrush; inability to raise his or her hand to his or her mouth; receiving treatment for an active dental or denture problem; or a diagnosis of dysphagia requiring thickened liquids.

### Randomization and Control of Cross-Contamination

Randomization will be concealed until after the initial screen and consents are obtained and an ID number is assigned to the subject. Dr. Kassab will prepare sealed envelopes to implement randomization. Inside each envelope will be the subject's group assignment, determined using a random number generator, with randomization to be conducted in blocks by nursing home site and time to ensure equal assignments across the two groups at the completion of the study and approximately equal assignments throughout the study to control for unknown temporal effects. While randomization by site is an acceptable method for controlling cross-contamination of conditions, we will randomize by individual. The rationales for this decision are, first, nursing homes are unstable environments[51] and second, nursing home quality indicators can fluctuate from one six-month period to the next[52]. Changes in quality indicators may reflect changes in resident profiles and/or changes in quality of care, both of which could affect the results of our study if randomization occurred at the facility level. Randomization by subject will provide greater power than randomization by NH.

Cross-contamination is a significant threat because control and experimental interventionists have the potential to interact during the provision of mouth care. Training for CRB raters, control interventionists, and experimental interventionists will occur separately. There will be no cross-training. All team members will be blinded to the specific aims of the study. All team members (CRB raters, interventionists) will be blinded to their assignment to the control or experimental arms of the study. Interventionists will be instructed to provide mouth care in private bathrooms with only the CRB rater present, to avoid having interventionists inadvertently observe each other. CRB raters will be paired exclusively with their control interventionists or with their experimental interventionists, to avoid bias in rating.

The potential exists for the control interventionists to address CRBs in some way by virtue of their experiences or humanistic approaches to care. While this potential cannot be ethically eradicated, its possible influence on the results can be addressed by having the control and experimental interventionists complete a "Provision of Mouth Care Form" at the conclusion of each mouth care interface. The "Provision of Mouth Care Form" will have a group of forced-choice questions concerning the completion of mouth care. There will be one open-ended question that requires the interventionists to describe what, if any, activities he or she employed to provide mouth care. An open-ended question is preferable to listing strategies or techniques, because the listing of these items could unblind the interventionist and threaten the design of the study. The experimental interventionists would be trained to complete the form by writing specific threat reduction strategies taught to them during their training. The control interventionists would be instructed to simply complete the form. By having both control and experimental interventionists complete the same form, additional risks for cross-contamination are reduced.

The PD will conduct random observations of 10% of the mouth care sessions provided by the control interventionists. The PD will complete a checklist of care-resistant strategies taught to the experimental interventionists. If the control interventionist is successfully employing these strategies to reduce CRB, the control interventionist will be debriefed, compensated for unworked scheduled shifts, and removed from the study.

### Treatment Fidelity

We will use several methods to monitor and enhance the reliability of our intervention[53]. Team members will be trained separately as either control interventionists and their CRB raters, or experimental interventionists and their CRB raters. Undergraduate and graduate nursing students will be hired and trained as experimental interventionists, control interventionists, or CRB raters. Training for the experimental interventionists consists of 3 hours of total training: best mouth care practices; explanation of dementia and theory behind threat-reduction techniques; detailed description of categories of CRB; strategies to recognize, prevent, and reduce CRB; and completion of specific instruments. The control interventionists receive 1 hour of didactic training regarding best mouth care practices and completion of specific instruments. The CRB raters receive 5 hours of training: 2 hours in the classroom and 3 hours in a NH. The first didactic hour addresses detailed descriptions of categories of CRB. The second didactic hour involves the correct use of the RTC-r and identifying type and quality of CRB from the training vide. The remaining 3 hours will occur in a NH during the baseline observations of mouth care and corresponding CRB data collection. The CRB raters will each complete one 3-hour period with either the PI or the project director (PD) during the 7-day observation period. At the conclusion of the 3-hour period, the CRB rater must have 90% agreement with either the PI or the PD; failure to reach 90% will result in retraining using the 2-hour didactic content and another 3-hour simultaneous data collection period until 90% is reached.

A treatment fidelity checklist will be used by the PD to monitor delivery of the intervention. The checklist contains components of the MOUTh intervention protocol, followed by "yes" (properly perfomed), "no" (not properly performed or incorrectly omitted), or n/a (not applicable). Ten percent (10%) of the mouth care sessions between experimental interventionists and NH subjects will be randomly rated. Retraining of the experimental interventionist will occur if any component of the MOUTh intervention protocol is not properly followed or is omitted. Additionally, experimental interventionists will complete the same treatment fidelity checklist if the MOUTh intervention was not delivered according to protocol. The PI and PD will monitor receipt of the intervention using measures for subject participation in the protocol: intervention dose (duration of mouth care session) and frequency (number of times mouth care completed). These methods and measures will help the team to monitor and ensure the reliability of the MOUTh intervention, its delivery, and its receipt.

### Instruments and Measures

The schedule for data collection is listed in Table 1 located at the end of the manuscript.

***Resistance to care ***is a dependent variable for specific aim #1 and hypothesis H1 and a mediating variable for specific aim #2 and hypothesis H2. It will be measured using a refinement of the Resistiveness to Care Scale, which was developed specifically for use with persons with dementia[54]. The original instrument listed 13 behaviors. Each behavior was rated according to duration and to intensity (mild, moderate, or extreme). The original instrument was designed to be used with videotapes; thus, each behavior was recorded once and the duration for all episodes was measured using time in categories (0 = none, 1-16 seconds = 1; 17-59 seconds = 2; 1-2 minutes = 3; more than 2 minutes = 4). The original RTC was developed and tested using 68 subjects at three sites (311 observations), with α = 0.82 and Kappa values ranging from 0.82 to 0.92[54].

In our pilot study, videotaping mouth care was problematic because of confined space issues (small bathrooms) and privacy concerns[35]. As noted in the pilot study,[35] the RTC was refined and tested for this study. The refinements included removing the duration component, measuring the intensity for each individual behavior, counting the frequency of each behavior within the intensity category, summing all episodes of CRB, and then standardizing the scores by dividing the sum with the duration of mouth care (in minutes) to obtain the rate of CRBs[35].

***Oral health ***of NH residents, a dependent variable attached to specific aim #2 and hypothesis H2, will be operationalized as the total score obtained from the Oral Health Assessment Tool [OHAT]. The OHAT is a modification of the Brief Oral Health Status Examination,[55] an oral health instrument developed specifically for NH residents with moderate to severe dementia. Each of the OHAT's eight categories is pertinent to specific oral structures and is scored from 0 (healthy) to 2 (unhealthy), resulting in a score ranging from 0-16. Internal consistency was obtained using test-retest percent agreements and Intra-carer and inter-carer correlation coefficients for total scores[56]. Intra-carer total OHAT scores achieved a correlation coefficient 0.78 (P < 0.001); inter-carer total OHAT scores achieved a correlation coefficient of 0.74 (P < 0.001). Validity was determined by comparing each of the eight categories with accepted dental criteria and instruments using clinical examinations by a qualified dentist[56]. Oral health assessors blinded to the specific aims of the study and subject assignment will collect this data per the schedule presented in Table 1.

***Functional status ***is a co-variate to CRB because CRB increases with higher levels of functional dependence[57,58]. This variable will be operationalized as the total score obtained from the Katz Activities of Daily Living (ADL) Index. The Katz ADL Index assesses overall performance in six areas of self-care: bathing, dressing, toileting, transferring, continence, and feeding[59]. A score of 1 (completely dependent), 2 (requires assistance) or 3 (completely independent) is assigned for each of the six areas, with scores ranging from 6 (completely dependent) to 18 (completely independent).

***Cognitive impairment ***is a co-variate to CRBs because CRB increases exponentially as cognitive impairment progresses[60]. This variable will be operationalized as a score of 2 or greater on the **Global Deterioration Scale (GDS)**. This instrument is used to quantify dementia severity by placing it in one of seven stages: (1) no cognitive decline-normal, 2) very mild cognitive decline-forgetfulness, (3) mild cognitive decline-early confusional, (4) moderate cognitive decline-late confusional, (5) moderately severe decline-early dementia, (6) severe cognitive decline-middle dementia, and (7) very severe cognitive decline-late dementia[61]. Other researchers have noted correlations from 0.88 - 0.91, P < .05 over six data collections with the Alzheimer's Disease Assessment Scale (ADAS)[62]. The data from the GDS will be used to identify relationships between the severity of cognitive decline and CRBs. The **Mini-Mental State Examination, MMSE**[49] will be used to determine capacity for consent and will be obtained once, during the consent process.

***Demographic data, co-morbidities***, and ***use of psychoactive medication ***are control variables that will be collected from the medical records at baseline. *Demographic data *are necessary for comparisons between control and experimental subjects. Co-morbidities will be collected using the *Charlson Co-morbidity Index*,[63] a weighted index that takes into account the number and seriousness of co-morbid diseases, to calculate a co-morbidity score for each subject. *Use of psychoactive medication *will be calculated from the medical records. Actual received dosages of regularly scheduled and "as needed" anti-depressant medication and anti-psychotic medication will be summed for each subject daily for the 7-day observation period and the 21-day intervention period. Both co-morbid diseases and psychoactive medication use may explain fluctuations in CRBs during the study.

***New infections***. Medical records will also be examined weekly for any new diagnosis of infection (e.g., urinary tract infection, pneumonia), and such infections will be recorded as bivariate data. This variable is a mediating variable for CRB. The "new infection" variable will be used as a control variable for RTC-r scores.

***Time ***is an independent variable that will be incorporated into statistical models to determine the effect of the intervention on CRB and oral health status over specified temporal intervals and shifts (day and evening).

***Dosage ***and ***frequency ***of the intervention are independent variables that will be incorporated into statistical models to determine the effect of the intervention on CRB and oral health status, and to compare the efficacy of the intervention to standard mouth care. Dosage will be calculated by the observer, using a stopwatch to measure the duration of each mouth care session. Dosage is also used to calculate the rate of care-resistance using the RTC-r. Frequency will be measured by counting the number of times mouth care was completed; percentage of completed mouth care will be calculated for each NH subject by dividing the number of successful mouth care completions by 42 (twice daily mouth care for 3 weeks).

### Describing the Cost of the Intervention

Specific Aim #3 is "Calculate the cost of the intervention." Describing the costs associated with the mouth care intervention and developing methods for documenting these costs are important for evaluating the public health impact of the intervention and provides the basis for future, more elaborate cost analysis work. Because this is a new intervention with little data describing its cost, activity-based costing methods will be used to determine the cost of the intervention prospectively. Costs that will be determined will include developmental and training costs, costs associated with identifying and enrolling participants, overhead, equipment and supply costs, and personnel expenses. Intervention costs will be dichotomized as being either fixed or variable: fixed costs do not vary with the number of patients treated and include the costs of office space, administrative support and equipment (such as computers or cell phones); variable costs do vary with the quantity of services provided and include personnel costs (time spent by caregivers in providing services) and supplies. For personnel costs, time spent by each staff member on activities related to the intervention will be tracked using activity logs, which will be checked regularly for accuracy. Total time spent on the intervention will then be multiplied by the hourly wage, including fringe benefits, to determine costs of personnel time. Intervention costs will include the costs of training the interventionists, but will exclude costs associated with research and monitoring, such as costs of training the CRB raters, time spent by CRB raters, and costs related to the randomization of study subjects.

### Procedures

Table 1 (at the end of the manuscript) illustrates the steps in the proposed study along with the data collection schedule.

### Statistical Analysis

Individual growth models using multilevel analysis will be used to estimate the efficacy of the intervention for reducing CRBs in persons with dementia, and to estimate the overall efficacy of the intervention using oral health outcomes. Multilevel models (also referred to as hierarchical linear models) will be used because repeated observations of participants are nested within nursing homes[48,64-68]. Multilevel models deal with the "unit of analysis" problem that face nested studies, that is the units of analysis are not independent, but instead the observations are clustered[48,66,69,70]. Because multiple observations are nested

within participants (i.e., repeated measures), individual growth models will be used. Among the advantages of using individual growth models are the ability to deal with an unequal number of measurement occasions among individuals and heterogeneity of variance[48,65,66,71].

Three-level multilevel models will be estimated for CRBs and oral health outcomes in which the first level is the date/time at which the behaviors or outcomes are observed; the second level is the participant; and the third level is the NH in which the participant resides. Explanatory variables to be included at the first level are dosage; frequency; time; and a dummy variable for baseline versus intervention. Explanatory variables to be included at the second level are a dummy variable for whether the participant is in the intervention group or control group; functional status; cognitive impairment; demographic data (gender, ethnicity, race, age, duration of stay in a nursing home); co-morbid conditions; and the use of psychoactive medications. Variables are discussed and defined above.

Describing the costs associated with the MOUTh intervention and developing methods for documenting these costs is important for evaluating the public health impact of the intervention. Because this is a new intervention with little data describing its cost, activity-based costing methods will be used. Determining the cost of the intervention will involve several steps. First, resources used to deliver the intervention will be determined.. These resources will include personnel, supplies and other expenses. Next, the amounts used of these resources will be documented. In the case of personnel time, the amount of time spent on activities related to the intervention will be determined using data from the RTC-r instrument, in which the exact amount of time (in seconds) the interventionists required to perform mouth care will be recorded. The amounts used of each resource will be multiplied by the unit cost of each resource to determine the cost associated with the resource. For personnel costs, the time spent by each experimental interventionist will be multiplied by the hourly compensation associated with CNAs. Finally, costs will be summed across all components of the intervention and divided by the number of NH residents treated to determine the average cost per NH resident of delivering the MOUTh intervention. These data will inform a future cost-effectiveness study.

## Discussion

Conceptualizing care-resistance as a fear response to the threat of mouth care brings a new and innovative slant to the MOUTh intervention proposed in this application. For the past 30 years, researchers and clinicians have grappled with methods to improve the oral health of NH residents. Those residents with dementia have been systematically excluded due to consent concerns or design decisions. A randomized clinical study that actively studies the effect of a behavioral intervention in this overlooked population has both great scientific and clinical potential.

We took several steps to strengthen the internal validity of this design. First, randomization at the individual level instead of cluster randomization removes any institutional artifact from the results. The use of outside interventionists, not NAs working in the NHs, removes another confounding variable, staffing levels. Attention to treatment fidelity is another important concern. Cross-contamination is a serious potential threat to the design of this study, and multiple strategies have been put in place to minimize this threat.

The potential for this intervention to radically and positively change the care received by persons with dementia is enormous. Its translational possibilities are exciting. We are encouraged by our pilot data and ready to undertake this project to improve care for persons suffering from the dual effects of dementia and poor oral health.

## Competing interests

The authors declare that they have no competing interests.

## Authors' contributions

RJ, AK, BT contributed to the overall conceptualization and design of the protocol. EM, CK, and DL contributed to the design of the protocol. EM participated in the revision of specific instruments used in the protocol. DL contributed to the development of specific aim #3 and all content related to that specific aim; and CK provided statistical support and conducted the power analyses. RJ drafted the manuscript which was critically revised by AK, BT, and EM. All authors read and approved the final manuscript.

## Pre-publication history

The pre-publication history for this paper can be accessed here:

http://www.biomedcentral.com/1472-6831/11/30/prepub
